# Supplementary material for: Intra‐lymph node crosslinking of antigen‐bearing polymers enhances humoral immunity and dendritic cell activation
Source: Bioeng Transl Med. 2024 Jul 17;9(6):e10705. doi: 10.1002/btm2.10705 (PMC11558197; doi:10.1002/btm2.10705)

**Video S1:** Demonstration of crosslinking at 7.5% w/v of 5 µL Tz-PEG and TCO-PEG.


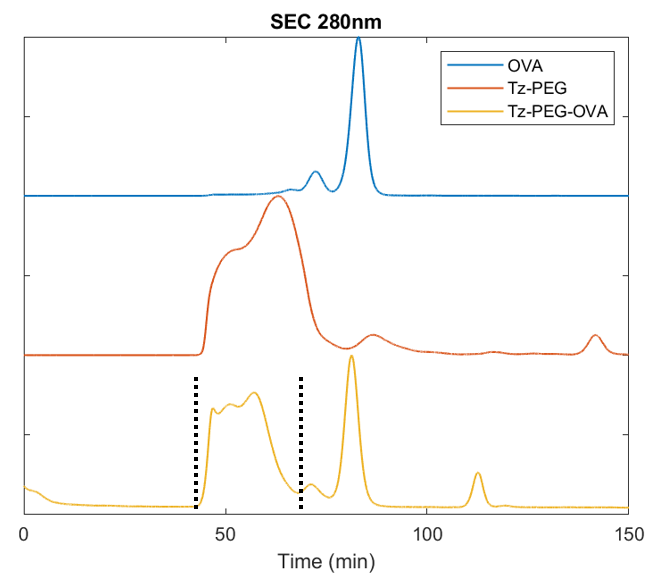


**Figure S1**: Size Exclusion Chromatography (SEC) chromatograms at 280 nm of OVA, PEG, and PEG-OVA to illustrate the purification method. The duration of material collection of PEG in the Tz-PEG-OVA group is shown by the dotted lines. All chromatograms were scaled to the same maximum intensity.


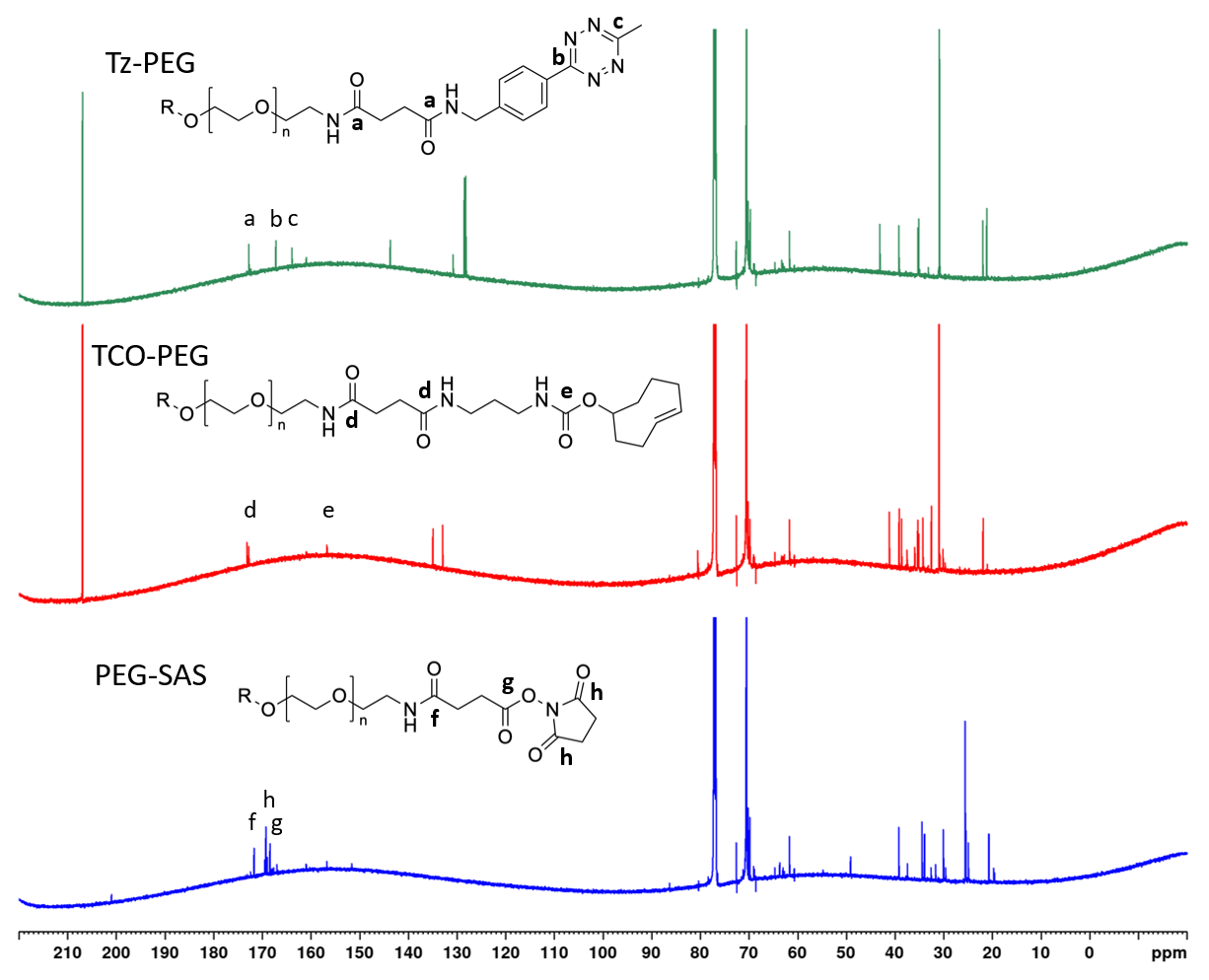


**Figure S2**: Carbon-13 nuclear magnetic resonance (CNMR) spectra of PEG before and after functionalization with Tz and TCO. A peak at 168 ppm (g) is present in unreacted PEG-SAS but disappears when PEG is reacted, replaced with a peak at 173 ppm (a,d) in Tz-PEG and TCO-PEG.


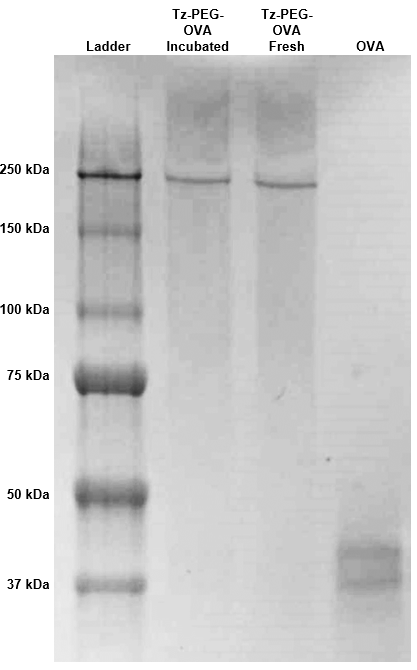


**Figure S3**: Sodium dodecyl sulfate–polyacrylamide gel electrophoresis (SDS-PAGE) gel showing the absence of unbound OVA in Tz-PEG-OVA, whether fresh (lane 3) or incubated in PBS at 37 °C for one week (lane 2). OVA when bound to Tz-PEG appears as a streak of staining through high molecular weights near the top of the gel.

**Figure S4**: Rheology time sweeps of mixed Tz-PEG and TCO-PEG materials at 3.75% (w/v) in water. (A) Measures of storage modulus (G’) and loss modulus (G’’) of the materials for five min immediately after mixing, demonstrating an increasing G’ as the crosslinking density increases. (B) After the experiment in (A), the geometry was lifted so that the materials could be stirred together for more complete mixing, and the time sweep was then performed again. After mixing, G’ was 8.7-fold higher than at the end of the experiment in (A), and G’ was consistently greater than G’’, demonstrating its activity as an elastic hydrogel.

**Figure S5**: Clearance of OVA from the injection site in the ankle, normalized to post-injection values (n=6).

**
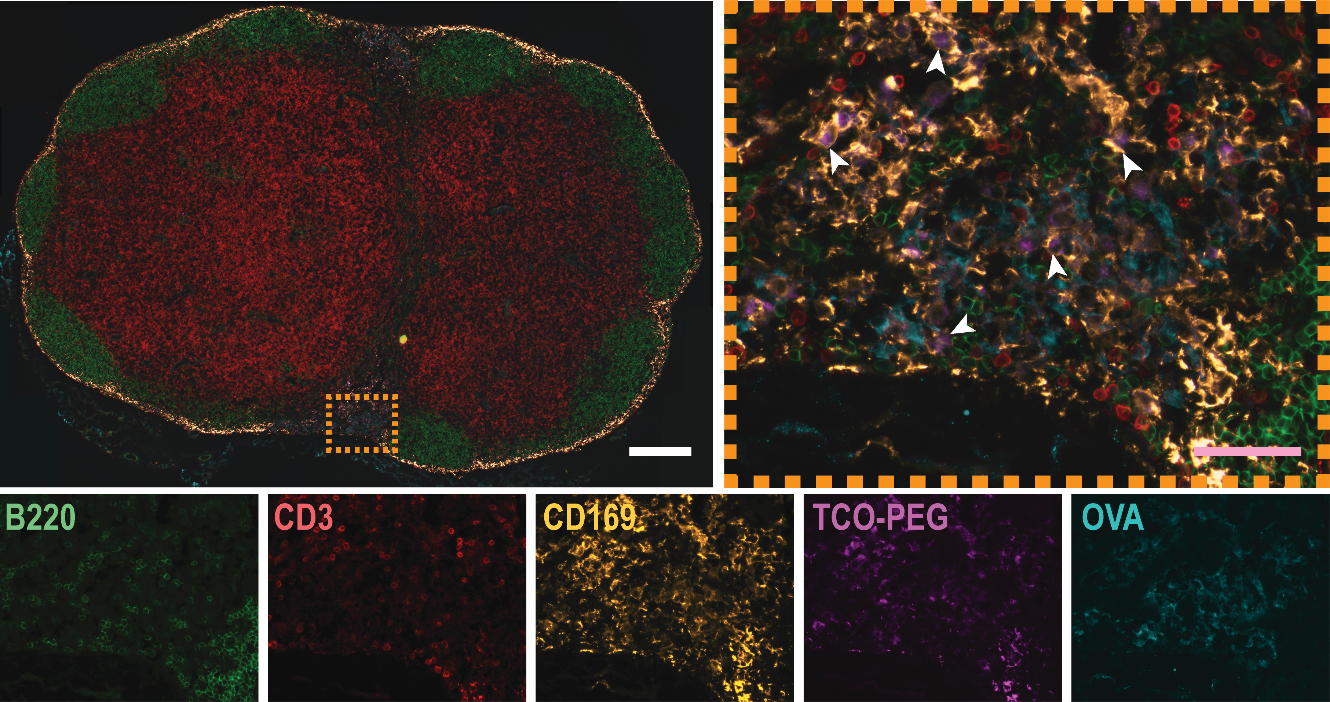
Figure S6**: Immunofluorescence staining of the PEG + Free OVA + “Click” group. Individual fluorescence channels for the zoomed image are shown below: Alexa Fluor 488 (B220, B cells), Spark YG 570 (CD3, T cells), Alexa Fluor 594 (CD169, macrophages), Cy5 (PEG-TCO), and DyLight 755 (OVA, amplified with anti-OVA antibody). White arrowheads note the apparent encapsulation of PEG-TCO by CD169^+^ macrophages. Note: some bleed-through was seen in the Cy5 channel of Alexa Fluor 594; when merged, the dimmer bleed-through into Cy5 is suppressed under Alexa Fluor 594, allowing true Cy5 signal to be visible. The white scale bar denotes 250 µm and the pink scale bar denotes 50 µm.


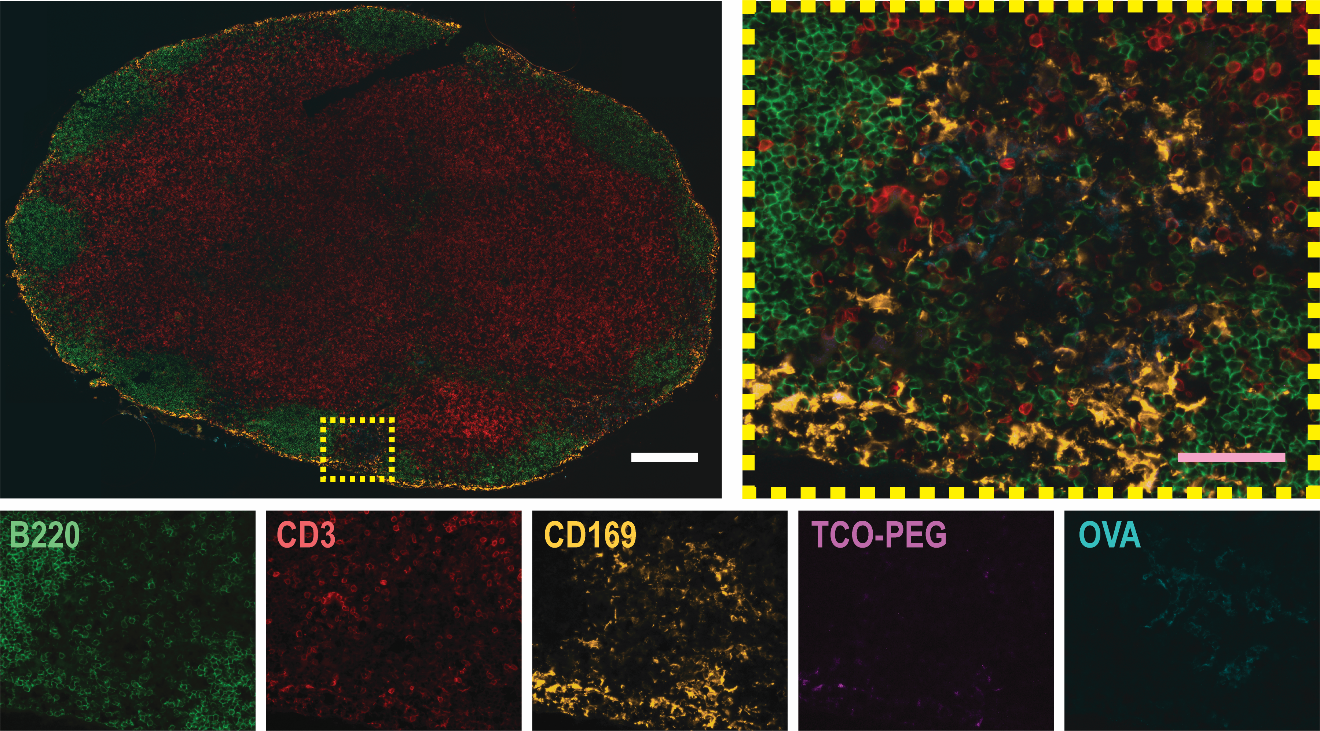


**Figure S7**: Immunofluorescence staining of the PEG-OVA group. Individual fluorescence channels for the zoomed image are shown below: Alexa Fluor 488 (B220, B cells), Spark YG 570 (CD3, T cells), Alexa Fluor 594 (CD169, macrophages), Cy5 (PEG-TCO), and DyLight 755 (OVA, amplified with anti-OVA antibody). Note: some bleed-through was seen in the Cy5 channel of Alexa Fluor 594; when merged, the dimmer bleed-through into Cy5 is suppressed under Alexa Fluor 594, allowing true Cy5 signal to be visible. The white scale bar denotes 250 µm and the pink scale bar denotes 50 µm.


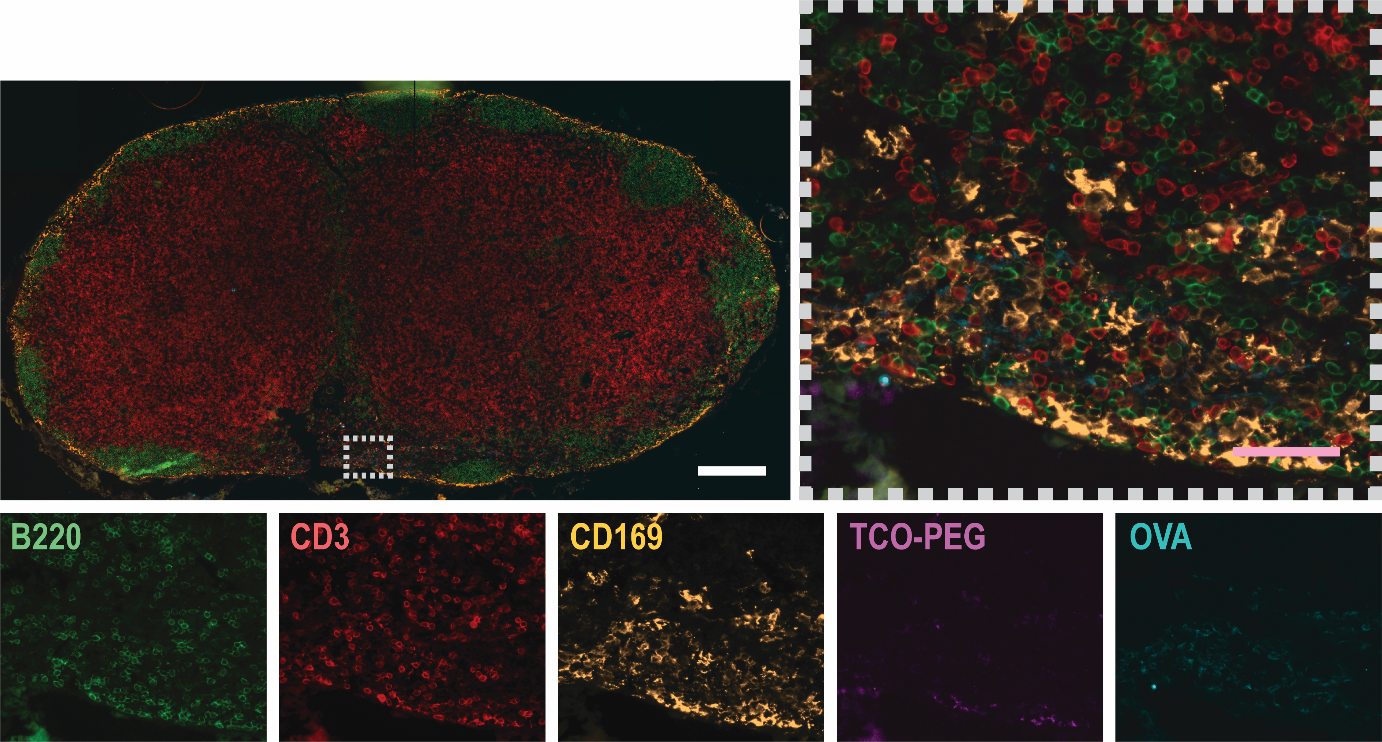


**Figure S8**: Immunofluorescence staining of the PEG + Free OVA group. Individual fluorescence channels for the zoomed image are shown below: Alexa Fluor 488 (B220, B cells), Spark YG 570 (CD3, T cells), Alexa Fluor 594 (CD169, macrophages), Cy5 (PEG-TCO), and DyLight 755 (OVA, amplified with anti-OVA antibody). Note: some bleed-through was seen in the Cy5 channel of Alexa Fluor 594; when merged, the dimmer bleed-through into Cy5 is suppressed under Alexa Fluor 594, allowing true Cy5 signal to be visible. The white scale bar denotes 250 µm and the pink scale bar denotes 50 µm.


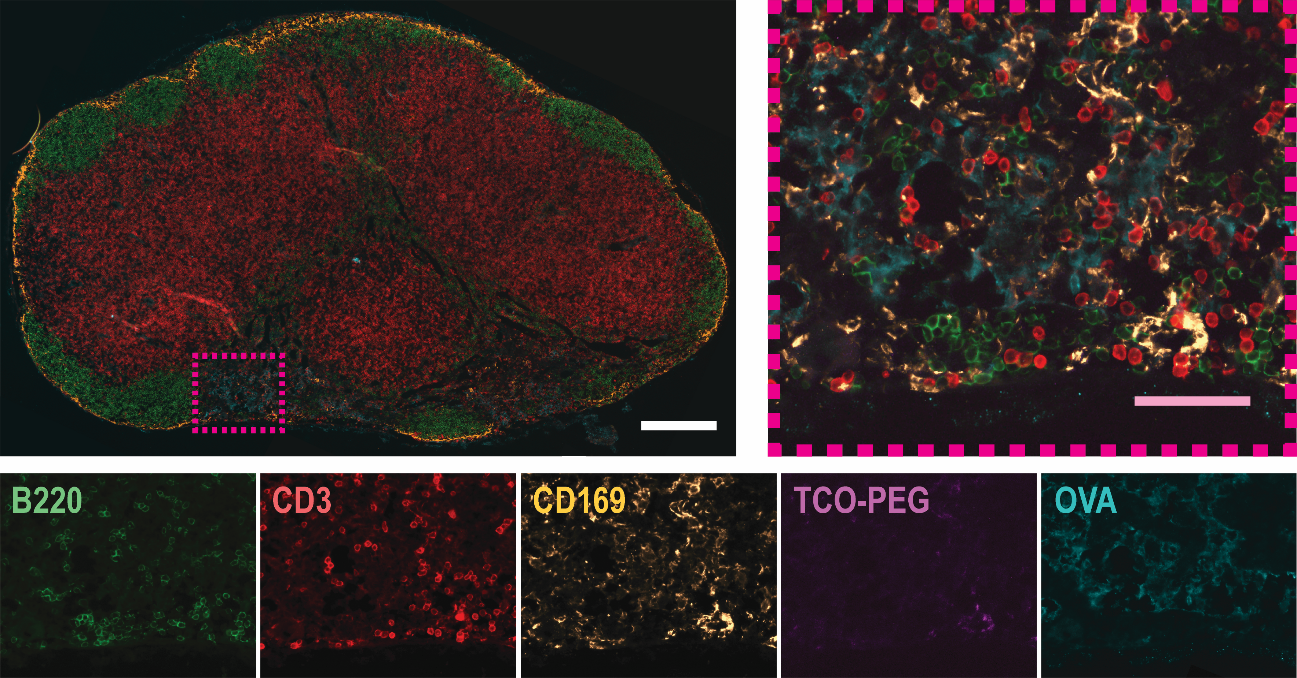


**Figure S9**: Immunofluorescence staining of the Alum + OVA group. Individual fluorescence channels for the zoomed image are shown below: Alexa Fluor 488 (B220, B cells), Spark YG 570 (CD3, T cells), Alexa Fluor 594 (CD169, macrophages), Cy5 (PEG-TCO), and DyLight 755 (OVA, amplified with anti-OVA antibody). Note: some bleed-through was seen in the Cy5 channel of Alexa Fluor 594; when merged, the dimmer bleed-through into Cy5 is suppressed under Alexa Fluor 594, allowing true Cy5 signal to be visible. The white scale bar denotes 250 µm and the pink scale bar denotes 50 µm.

**Figure S10**: Antibody subclass analysis (n=6). Alum + OVA is significantly greater than the PEG-OVA + “Click” and PEG + Free OVA + “Click” groups in all subclasses.

**Table S1**: Antibodies used for immunofluorescence staining of lymph node sections.


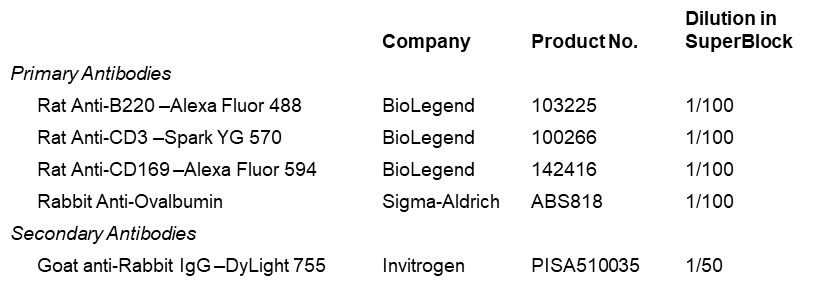


**Table S2**: Antibodies and stains used for flow cytometry.


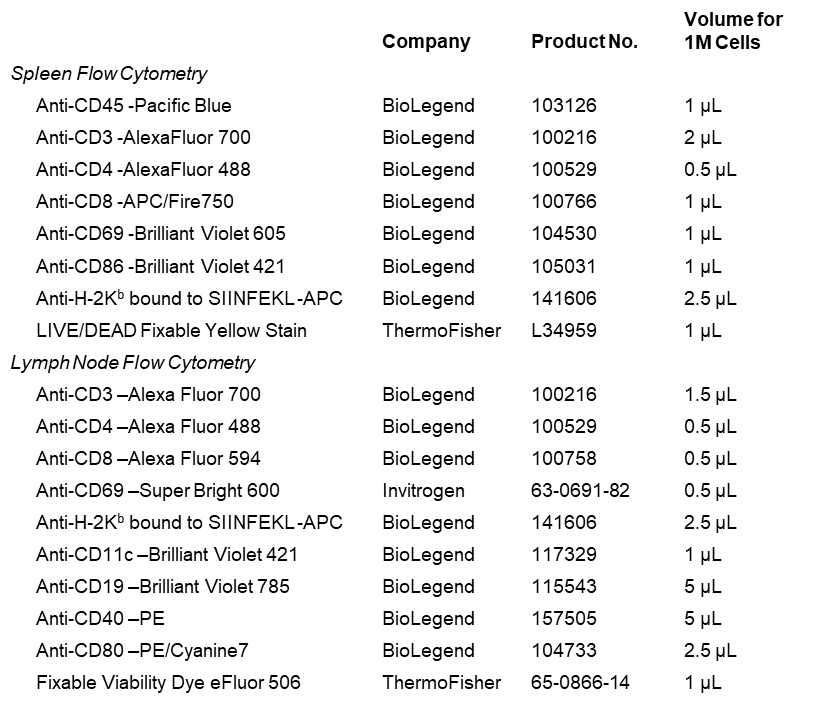

Supplement: Supplementary file 1 — FIGURE S1: Size exclusion chromatography (SEC) chromatograms at 280 nm of OVA, PEG, and PEG–OVA to illustrate the purification method. The duration of material collection of PEG in the Tz–PEG–OVA group is shown by the dotted lines. All chromatograms were scaled to the same maximum intensity. FIGURE S2: Carbon‐13 nuclear magnetic resonance (CNMR) spectra of PEG before and after functionalization with Tz and TCO. A peak at 168 ppm (g) is present in unreacted PEG–SAS but disappears when PEG is reacted, replaced with a peak at 173 pm (a,d) in Tz–PEG and TCO–PEG. FIGURE S3: Sodium dodecyl sulfate–polyacrylamide gel electrophoresis (SDS–PAGE) gel showing the absence of unbound OVA in Tz–PEG–OVA, whether fresh (lane 3) or incubated in PBS at 37°C for one week (lane 2). OVA when bound to Tz–PEG appears as a streak of staining through high molecular weights near the top of the gel. FIGURE S4: Rheology time sweeps of mixed Tz–PEG and TCO–PEG materials at 3.75% (w/v) in water. (a) Measures of storage modulus (G′) and loss modulus (G″) of the materials for 5 min immediately after mixing, demonstrating an increasing G′ as the crosslinking density increases. (b) After the experiment in (a), the geometry was lifted so that the materials could be stirred together for more complete mixing, and the time sweep was then performed again. After mixing, G′ was 8.7‐fold higher than at the end of the experiment in (a), and G′ was consistently greater than G″, demonstrating its activity as an elastic hydrogel. FIGURE S5: Clearance of OVA from the injection site in the ankle, normalized to post‐injection values (n = 6). FIGURE S6: Immunofluorescence staining of the PEG + Free OVA + “Click” group. Individual fluorescence channels for the zoomed image are shown below: Alexa Fluor 488 (B220, B cells), Spark YG 570 (CD3, T cells), Alexa Fluor 594 (CD169, macrophages), Cy5 (PEG–TCO), and DyLight 755 (OVA, amplified with anti‐OVA antibody). The white arrowheads note the apparent encapsulation of PE [file BTM2-9-e10705-s001.docx]
